# Zebrafish transgenic Enhancer TRAP line database (ZETRAP)

**DOI:** 10.1186/1471-213X-6-5

**Published:** 2006-02-14

**Authors:** Benjamin GH Choo, Igor Kondrichin, Sergey Parinov, Alexander Emelyanov, William Go, Wei-chang Toh, Vladimir Korzh

**Affiliations:** 1 Institute of Molecular and Cell Biology, 61 Biopolis Dr., Proteos, 138673, Singapore; 2 Nanyang Polytechnic, 180 Ang Mo Kio Avenue 8, 569830, Singapore; 3 Department of Biological Sciences, National University of Singapore, 14 Science Drive 4, 117543, Singapore; 4 Temasek Life Science Laboratory, 1 Research Link, 117604, Singapore

## Abstract

**Background:**

The zebrafish, *Danio rerio*, is used as a model organism to study vertebrate genetics and development. An effective enhancer trap (ET) in zebrafish using the *Tol2 *transposon has been demonstrated. This approach could be used to study embryogenesis of a vertebrate species in real time and with high resolution.

**Description:**

The information gathered during the course of systematic investigation of many ET transgenic lines have been collected and compiled in the form of an online database – the Zebrafish Enhancer TRAP lines database (ZETRAP).

**Conclusion:**

ZETRAP is a web-based system that provides data and information to the scientific community about the developmental, genetic and genomic aspects of transgenic zebrafish lines obtained using *Tol2 *transposon-mediated transgenesis. The current version (version 1.0) contains description of 27 ET lines that express EGFP in various organs and tissues, for example, heart, brain, notochord, gut, etc. It also includes information on insertion sites of the *Tol2 *transposon in these lines.

## Background

Zebrafish is a small tropical ornamental fish whose attributes such as optical transparency of embryos, short generation time and availability of mutants make it a favorable model for vertebrate developmental biology [1]. In addition, the embryonic development of zebrafish is relatively well-characterized and its large-scale husbandry well-established [2,3]. Hence, the zebrafish has been used as a convenient and effective bioassay for analyzing both gene function and regulation [4].

The post-genomic era has led to the progression of functional studies and spurred the development of several online databases, such as FlyBase for *Drosophila *[5], MGI for mouse [6] and ZFIN for zebrafish [7], containing various information about these model animals. We also see an emergence of more specialized databases, such as the database of duplicated genes [8] or genetic screen database [9]. Taken together, these databases represent a very informative and organized library, which provides the scientific community with necessary information.

Here, we introduce a new database of zebrafish transgenic lines, ZETRAP. It contains descriptions of transgenic lines generated using an enhancer trap system with modified *Tol2 *transposon from medaka [10]. In that article, only a handful of ET lines were described in sufficient detail. Other lines were presented rather superficially and the limited data available at that time was simplistically organized in a table. After this publication, the DNA constructs used in this study, and several transgenic lines were sent into many laboratories. Our analysis of requests for ET lines has indicated that the most popular lines are those described in more detail [10]. And yet our library of ET lines contains many other interesting lines that could also be very useful for researchers within the community. In line with such observation, we have made an effort to describe their GFP expression patterns in detail and in addition, provide other information of all published ET lines in a convenient format of an online database.

## Construction and content

The database has been compiled using Microsoft Office Publisher 2003. It can be browsed using Microsoft^® ^Internet Explorer 6.0 with Windows XP or Safari^® ^1.3 with OS10. Note that when using Safari^® ^to browse the "Gene Expression" section for pages describing individual lines, click on the image instead of the "more details" hyperlink; which is active only under Microsoft^® ^Internet Explorer environment.

**Figure 1:**
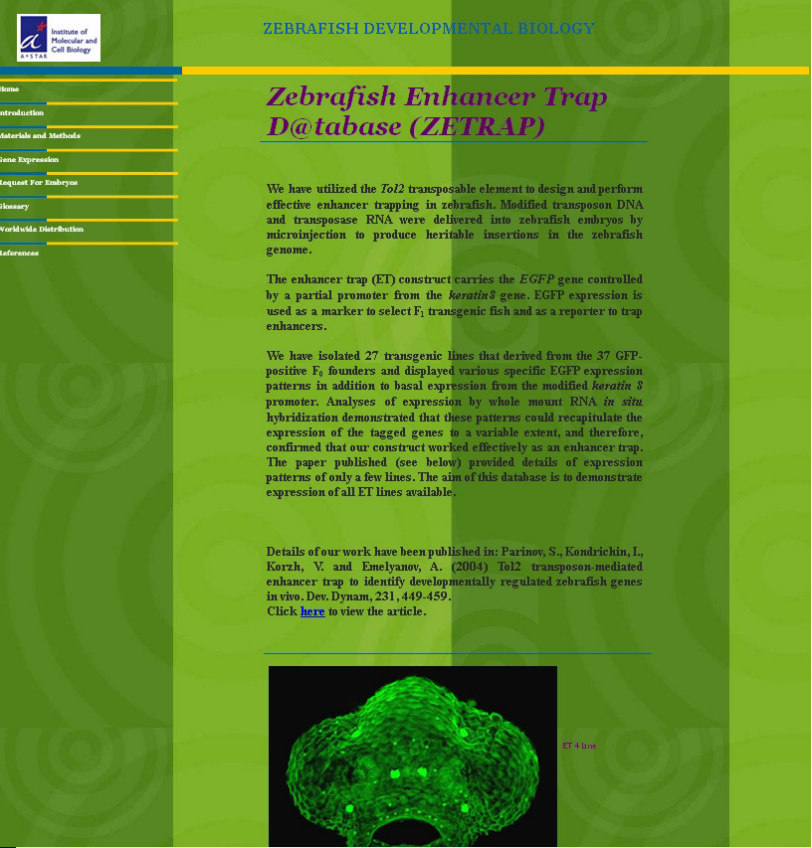
ZETRAP homepage is kept simple for easy navigation.

**Figure 2:**
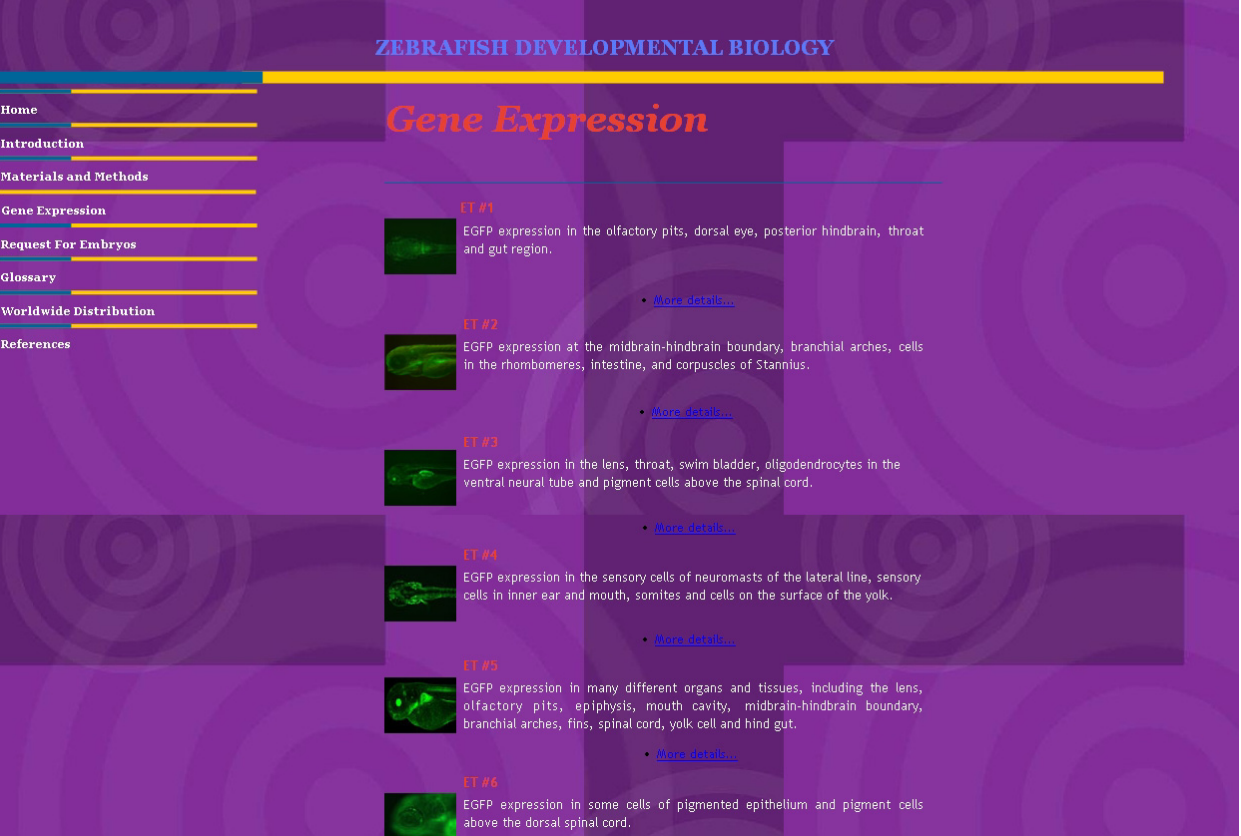
The Gene Expression view lists all ET lines and provides their brief description.

**Figure 3:**
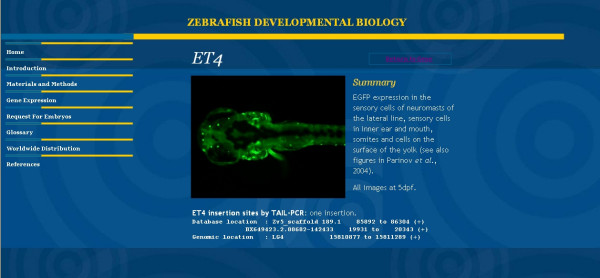
ET line view provides a low resolution image of expression pattern, brief description of expression pattern and insertion site.

**Figure 4:**
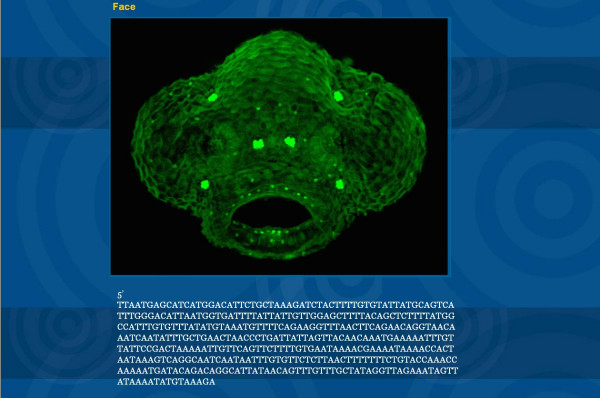
ET line view also contains several high resolution images and ends by showing TAIL-PCR data of sequences flanking the insertion sites.

**Figure 5:**
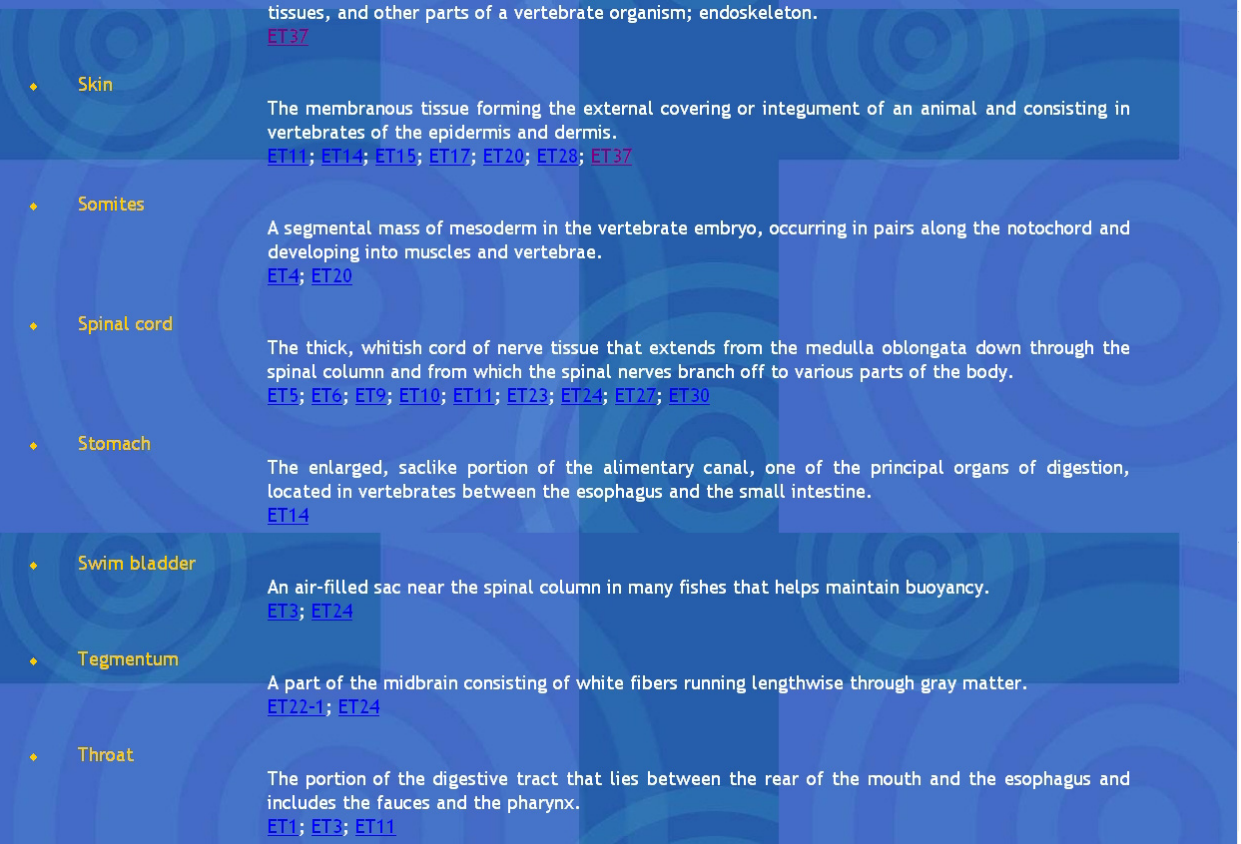
The Glossary view provides a list of anatomical terms used. These are hyperlinked to the ET lines containing GFP expression in these tissues and organs.

The current version of ZETRAP contains description of 27 ET transgenic lines (Figure 1 &2). Each transgenic line is allocated a page consisting of digital photos of the fluorescent larvae (Figure 3 &4), a brief summary of the EGFP expression phenotype, genomic DNA sequences flanking the insertion sites and genomic localization of inserts. Additional features of the database include a short introduction, a glossary section with links to related ET lines (Figure 5), worldwide distribution of these ET lines, materials and methods, and information on requesting these lines.

## Utility

In our screen we detected various specific EGFP expression patterns in addition to the basal skin-specific expression attributed to activity of the *keratin8 *minipromoter. The EGFP expression patterns were tracked up to various stages of development. Morphological studies of expression patterns were carried out using the compound Zeiss Axiophot2 microscope equipped with the digital camera Axiocam HRc (Zeiss, Germany). Most of the lines display characteristic expression pattern from 3 dpf, even though low level of basal GFP expression can be observed as early as 1 hpf. In contrast, ET2 for example, exhibits strong EGFP expression in the early stages due to the presence of maternal EGFP.

All recognizable anatomical structures defined by EGFP expression patterns were summarized in the "Glossary" section. This provides users with fast access to pages describing particular lines, which exhibit the expression pattern of interest and facilitates the selection process of EGFP expression in specific organs. It also simplifies the search process and provides information about the lines expressing similar expression patterns.

Besides providing descriptions of expression patterns of the transgenic lines, TAIL-PCR data of sequences flanking the insertion sites are also included. This provides a potential link of a particular gene(s) to specific aspects of development. If founding lines carried more than one insertion, where possible these insertions were linked to specific expression patterns.

## Discussion

One of the major aims of ZETRAP is to consolidate and organize information about ET lines into an accessible database on the worldwide web. Our analysis demonstrated that most requests that we have received were for those ET lines which expression patterns were presented in more details in the original publication [10]. We therefore realized that since a detailed description of GFP expression patterns in other ET lines were not included, researchers could have found it difficult to realize the potential of other less described ET lines for developmental studies. Thus this database provides information about GFP expression patterns of all ET lines described in [10]. We hope that this could improve the circulation of the ET lines and thus provide scientists with new research tools to carry out tissue or organ-specific developmental studies.

Transgenic animals are useful tools for developmental studies. This is also true for transgenic zebrafish, whereby the external embryonic development and transparency of embryos offer a new high resolution *in vivo *approach to anatomical investigation. After all, transgenic zebrafish lines (including ET lines) allow direct and detailed observation of GFP expression in various tissues and organs throughout embryogenesis even during later larval and adult development [11] (our unpublished results). In addition, the ET lines provide a useful tool for identification of tissue-specific enhancers. The transgenic lines described in ZETRAP therefore present a sensitive instrument of organogenesis studies. The stability of EGFP expression allows studies of live embryos throughout embryogenesis at high resolution. Together, these make the database an important starting point for planning such studies.

During systematic screening of ET lines, we have recorded more than 1000 high-resolution digital images, some of which are being used in this database. Currently, expression pattern of each ET line is illustrated by 3 to 5 images taken at various perspective views and optical zoom levels. Future version of this database could incorporate a more detailed time-based series of pictures, which will allow the user to track the emergence of expression in each ET line at the GFP and mRNA levels. Movies reflecting dynamic changes of expression patterns will be generated eventually, following distribution of these lines within the community. These additions to ZETRAP will further augment the analysis of organogenesis and better facilitate the correlation of individual gene expression. To develop the database further and provide a wider choice of transgenic lines, we continue our efforts in expanding this collection of ET lines in order to yield more phenotypes. These efforts would improve the usefulness of the database as a starting point for planning of developmental studies.

During the preparation of this manuscript we were informed about a related database [12]. The ZETRAP website is available via the World Wide Web at [13]. Questions regarding the ZETRAP should be addressed to Dr. Vladimir Korzh: vlad@imcb.a-star.edu.sg.

## Conclusion

Enhancer trap (ET) lines are transgenic zebrafish generated using *Tol2 *transposon-mediated trangenesis. ZETRAP is a web-based system intended to profile our current collection of 27 such ET lines. Tissues and organs-specific GFP expression are displayed in high-resolution digital images. ET lines can be utilized as a sensitive instrument of organogenesis studies. The stability of EGFP expression allows studies of live embryos throughout embryogenesis at high resolution. Together, these make the database an important starting point for planning such studies.

## Availability and requirement

The URL for current version of ZETRAP (ver. 1.0) is . The minimum internet browser requirement is Microsoft^® ^Internet Explorer 6.0 with Windows XP or Safari^® ^1.3 with OS10. For information on request of ET lines, please refer to the website.

## Authors' contributions

BGHC – made systematic microscopic analysis of expression patterns, designed the database and wrote the manuscript; IK – screened for ET lines and was involved in their molecular and morphological characterization with emphasis on lines that carry multiple insertions; SP – developed the concept of enhancer trap screen, designed constructs, generated transgenic lines, screened them and made their initial molecular and microscopic analysis; AE – developed the concept of enhancer trap screen, generated transgenic lines, screened them and made their initial molecular and microscopic analysis; WG – involved in molecular and morphological characterization of lines that carry multiple insertions, maintained the collection of ET lines and database; WCT – made systematic microscopic analysis of expression patterns; VK – developed the concept of enhancer trap screen, characterized expression patterns, modified the database, wrote and approved the manuscript.
